# Potassium bis­[bis­(1-benzyl-3-methyl­imidazolium)silver(I)] tris­(hexa­fluoridophosphate)

**DOI:** 10.1107/S1600536810051925

**Published:** 2010-12-18

**Authors:** Rosenani A. Haque, Abbas Washeel Salman, Choong Kah Whai, Ching Kheng Quah, Hoong-Kun Fun

**Affiliations:** a School of Chemical Sciences, Universiti Sains Malaysia, 11800 USM, Penang, Malaysia; b X-ray Crystallography Unit, School of Physics, Universiti Sains Malaysia, 11800 USM, Penang, Malaysia

## Abstract

In the title compound, K[Ag(C_11_H_12_N_2_)_2_]_2_(PF_6_)_3_, the 12-coordinate potassium cation lies on a crystallographic twofold axis and one of the hexa­fluoro­phosphate anions is generated by 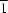 symmetry. In the complex cation, the Ag^I^ ion is coordinated by two C atoms; the two imidazolium rings are orientated at a dihedral angle of 8.14 (14)°. In the 1-benzyl-3-methyl­imidazolium units, the dihedral angles between imidazolium and phenyl rings are 80.47 (15) and 76.53 (14)°. The F atoms of the general-position hexa­fluoro­phosphate anion are disordered over two sets of sites in a 0.767 (17):0.233 (17) ratio. In the crystal, the hexa­fluoro­phosphate anions link the cations into three-dimensional networks *via* inter­molecular C—H⋯F hydrogen bonds and are further consolidated by π–π stacking [centroid–centroid distances = 3.5518 (15) Å] inter­actions.

## Related literature

For general background to and the biological activity of carbene derivatives, see: Lee *et al.* (2001 ▶); Bourissou *et al.* (2000 ▶); Herrmann & Köcher (1997 ▶); Hermann *et al.* (1996 ▶); Zhou *et al.* (2008 ▶); Wang & Lin (1998 ▶); Lin & Vasam (2007 ▶); Ray *et al.* (2007 ▶); Özdemir *et al.* (2010 ▶); Medvetz *et al.* (2008 ▶). For the stability of the temperature controller used in the data collection, see: Cosier & Glazer (1986 ▶). For related structures, see: Haque *et al.* (2010*a* ▶,*b* ▶). For bond-length data, see: Allen *et al.* (1987 ▶).
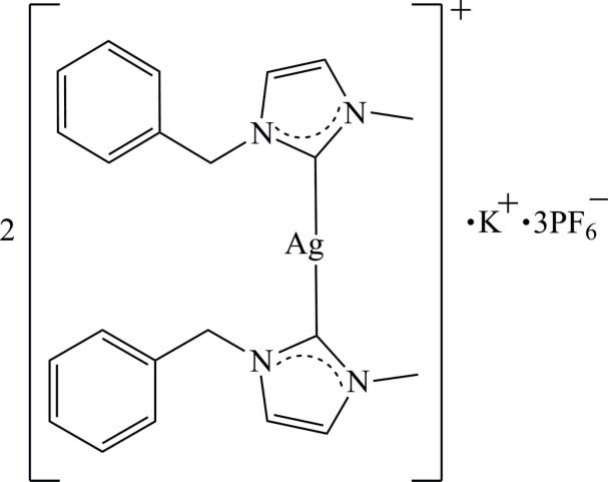


## Experimental

### 

#### Crystal data

K[Ag(C_11_H_12_N_2_)_2_]_2_(PF_6_)_3_
*M* *_r_* = 1378.65
Monoclinic, 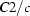

*a* = 19.917 (2) Å
*b* = 23.047 (2) Å
*c* = 11.5787 (12) Å
β = 103.108 (3)°
*V* = 5176.4 (9) Å^3^
*Z* = 4
Mo *K*α radiation
μ = 1.04 mm^−1^
*T* = 100 K
0.49 × 0.42 × 0.17 mm

#### Data collection

Bruker SMART APEXII DUO CCD diffractometer
Absorption correction: multi-scan (*SADABS*; Bruker, 2009 ▶) *T*_min_ = 0.632, *T*_max_ = 0.847
66454 measured reflections
7528 independent reflections
7058 reflections with *I* > 2σ(*I*)
*R*_int_ = 0.050

#### Refinement

*R*[*F* ^2^ > 2σ(*F* ^2^)] = 0.035
*wR*(*F* ^2^) = 0.124
*S* = 1.11
7528 reflections
372 parameters
51 restraints
H-atom parameters constrained
Δρ_max_ = 1.68 e Å^−3^
Δρ_min_ = −0.91 e Å^−3^

### 

Data collection: *APEX2* (Bruker, 2009 ▶); cell refinement: *SAINT* (Bruker, 2009 ▶); data reduction: *SAINT*; program(s) used to solve structure: *SHELXTL* (Sheldrick, 2008 ▶); program(s) used to refine structure: *SHELXTL*; molecular graphics: *SHELXTL*; software used to prepare material for publication: *SHELXTL* and *PLATON* (Spek, 2009 ▶).

## Supplementary Material

Crystal structure: contains datablocks global, I. DOI: 10.1107/S1600536810051925/hb5767sup1.cif

Structure factors: contains datablocks I. DOI: 10.1107/S1600536810051925/hb5767Isup2.hkl

Additional supplementary materials:  crystallographic information; 3D view; checkCIF report

## Figures and Tables

**Table 1:** Selected bond lengths (Å)

Ag1—C12	2.092 (2)
Ag1—C1	2.093 (2)

**Table 2:** Hydrogen-bond geometry (Å, °)

*D*—H⋯*A*	*D*—H	H⋯*A*	*D*⋯*A*	*D*—H⋯*A*
C14—H14*A*⋯F1^i^	0.93	2.45	3.285 (5)	149
C15—H15*A*⋯F6	0.97	2.51	3.204 (5)	129
C15—H15*B*⋯F4^i^	0.97	2.51	3.415 (7)	156
C22—H22*A*⋯F6^ii^	0.96	2.42	3.171 (5)	135

Symmetry codes: (i) 
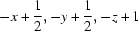
; (ii) 
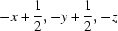
.

**Fig. 1.**
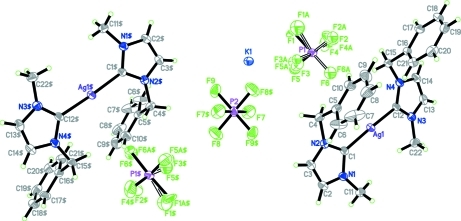
The molecular structure of (I) showing 50% probability displacement ellipsoids for non-H atoms. Both major and minor components of disorder are shown. Symmetry code: ($) -x, -y, -z. Coordination interactions between K cation and hexafluorophosphate anions have been omitted for clarity.

**Fig. 2.**
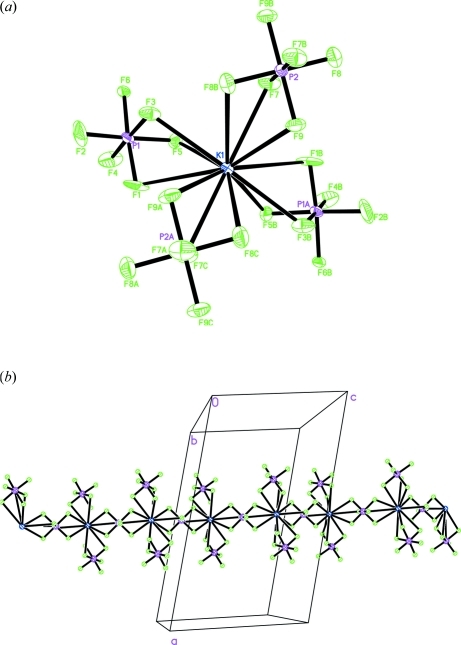
(a) Part of the crystal packing, highlighting the coordination environment for K cation. Displacement ellipsoids are drawn at the 30% probability level. Only major component of disorder is shown. (b) Extended coordination environment for K cations. Only major component of disorder is shown.

**Fig. 3.**
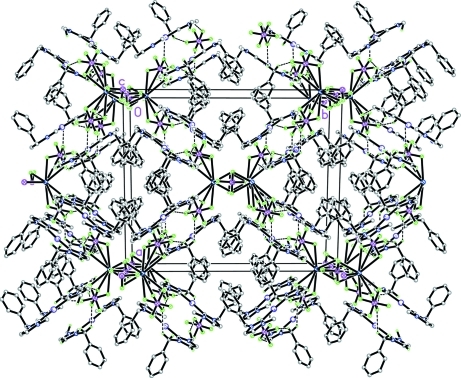
The crystal structure of (I), viewed along the c axis. H atoms not involved in hydrogen bonds (dashed lines) have been omitted for clarity. Only major component of disorder is shown.

## supplementary crystallographic information

### Comment

Metal complexes of carbenes based on imidazol-2-ylidene have received much attention in the past few years (Lee *et al.*, 2001). A major reason is that these *N*-heterocyclic carbenes(NHCs) can stabilize both high and low oxidation state metal ions and form stable complexes with a wide range of metals (Bourissou *et al.*, 2000; Herrmann & Köcher, 1997; Hermann *et al.*, 1996). Among these complexes, the family of silver NHC complexes have been receiving continuous attention (Zhou *et al.*, 2008). The *in-situ* deprotonation method using Ag_2_O as a basic metal source developed by Lin and co-workers (Wang & Lin, 1998) has been widely employed to synthesise these Ag-NHC complexes. The silver complexes act as effective carbene transfer agents to other metals and are much more stable than the free carbene (Lin & Vasam, 2007). Biological activity of many Ag-NHC complexes as antimicrobial and antitumour agents has been confirmed (Ray *et al.*, 2007; Özdemir *et al.*, 2010; Medvetz *et al.*, 2008).

The asymmetric unit of the title compound, (I), contains a bis(1-benzyl-3-methylimidazolium)silver(I) cation, a K cation and one and a half hexafluorophosphate anions (Fig. 1). The two imidazolium rings (N1/N2/C1-C3 and N3/N4/C12-C14) make a dihedral angle of 8.14 (14)°, indicating that they are almost parallel to each other. In the two 1-benzyl-3-methylimidazolium moieties [N1/N2/C1-C11 (*A*) and N3/N4/C12-C22 (*B*)], the dihedral angles between imidazolium and phenyl rings are 80.47 (15) and 76.53 (14)° in *A* and *B*, respectively. The P2 atom of the hexafluorophosphate anion is lying on a crystallographic inversion center (symmetry code: -x, -y, -z) whereas the 12-coordinate potassium cation (Fig. 2) lies on a crystallographic twofold axis (symmetry code: -x, y, -z+1/2). Six fluorine atoms (F1A-F6A) of the hexafluorophosphate anion are disordered over two positions with refined site-occupancies of 0.767 (17) : 0.233 (17). Bond lengths and angles are within normal ranges, and comparable to closely related structures (Haque *et al.*, 2010*a*,*b*)

In the crystal structure, (Fig. 3), the hexafluorophosphate anions link the cations into three-dimensional networks *via* intermolecular C14–H14A···F1, C15–H15A···F6, C15–H15BA···F4 and C22–H22A···F6 hydrogen bonds (Table 2) and are further consolidated by π-π stacking interactions between N1/N2/C1-C3 (centroid Cg1) and N3/N4/C12-C14 (centroid Cg2) rings, with a Cg1···Cg2 distance of 3.5518 (15) Å.

### Experimental

To a stirred solution of 1-benzyl-3-methylimidazolium hexafluorophosphate (0.5 g, 1.57 mmol) in acetonitrile (40 ml), Ag_2_O (0.37 g, 1.6 mmol) was added. The mixture was refluxed at 70 °C for 18 h in glassware wrapped in aluminum foil to exclude the light. The mixture was filtered through celite to remove excess Ag_2_O and the solvent was evaporated under vacuum. The white residue was washed with diethyl ether (2 × 3 ml) to afford the complex as a white powder, the yield was 0.66 g, 70.3%, *m. p.* = 421-423 K. Colourless blocks of (I) were obtained by slow diffusion of diethyl ether into a solution of the complex in acetonitrile at ambient temperature.

### Refinement

All H atoms were positioned geometrically and refined using a riding model with C–H = 0.93-0.97 Å and *U*_iso_(H) = 1.2 or 1.5 *U*_eq_(C). A rotating-group model was applied for the methyl groups. Six fluorine atoms (F1A-F6A) of the hexafluorophosphate anion are disordered over two positions with refined site-occupancies of 0.767 (17) : 0.233 (17). The minor component of disorder was refined isotropically and subjected to rigid bond and similarity restraints. The highest residual electron density peak is located at 0.82 Å from Ag1 and the deepest hole is located at 0.69 Å from Ag1.

### Crystal data

**Table:**

K[Ag(C_11_H_12_N_2_)_2_]_2_(PF_6_)_3_	*F*(000) = 2752
*M**_r_* = 1378.65	*D*_x_ = 1.769 Mg m^−^^3^
Monoclinic, *C*2/*c*	Mo *K*α radiation, λ = 0.71073 Å
Hall symbol: -C 2yc	Cell parameters from 9934 reflections
*a* = 19.917 (2) Å	θ = 2.5–37.7°
*b* = 23.047 (2) Å	µ = 1.04 mm^−^^1^
*c* = 11.5787 (12) Å	*T* = 100 K
β = 103.108 (3)°	Block, colourless
*V* = 5176.4 (9) Å^3^	0.49 × 0.42 × 0.17 mm
*Z* = 4	

### Data collection

**Table:**

Bruker SMART APEXII DUO CCD diffractometer	7528 independent reflections
Radiation source: fine-focus sealed tube	7058 reflections with *I* > 2σ(*I*)
graphite	*R*_int_ = 0.050
φ and ω scans	θ_max_ = 30.0°, θ_min_ = 1.4°
Absorption correction: multi-scan (*SADABS*; Bruker, 2009)	*h* = −28→27
*T*_min_ = 0.632, *T*_max_ = 0.847	*k* = −32→32
66454 measured reflections	*l* = −16→16

### Refinement

**Table:**

Refinement on *F*^2^	Primary atom site location: structure-invariant direct methods
Least-squares matrix: full	Secondary atom site location: difference Fourier map
*R*[*F*^2^ > 2σ(*F*^2^)] = 0.035	Hydrogen site location: inferred from neighbouring sites
*wR*(*F*^2^) = 0.124	H-atom parameters constrained
*S* = 1.11	*w* = 1/[σ^2^(*F*_o_^2^) + (0.072*P*)^2^ + 12.8353*P*] where *P* = (*F*_o_^2^ + 2*F*_c_^2^)/3
7528 reflections	(Δ/σ)_max_ = 0.001
372 parameters	Δρ_max_ = 1.68 e Å^−^^3^
51 restraints	Δρ_min_ = −0.91 e Å^−^^3^

### Special details

**Table:**

Experimental. The crystal was placed in the cold stream of an Oxford Cyrosystems Cobra open-flow nitrogen cryostat (Cosier & Glazer, 1986) operating at 100.0 (1) K.
Geometry. All esds (except the esd in the dihedral angle between two l.s. planes) are estimated using the full covariance matrix. The cell esds are taken into account individually in the estimation of esds in distances, angles and torsion angles; correlations between esds in cell parameters are only used when they are defined by crystal symmetry. An approximate (isotropic) treatment of cell esds is used for estimating esds involving l.s. planes.
Refinement. Refinement of F^2^ against ALL reflections. The weighted R-factor wR and goodness of fit S are based on F^2^, conventional R-factors R are based on F, with F set to zero for negative F^2^. The threshold expression of F^2^ > 2sigma(F^2^) is used only for calculating R-factors(gt) etc. and is not relevant to the choice of reflections for refinement. R-factors based on F^2^ are statistically about twice as large as those based on F, and R- factors based on ALL data will be even larger.

### Fractional atomic coordinates and isotropic or equivalent isotropic displacement parameters (Å^2^)

**Table:**

	*x*	*y*	*z*	*U*_iso_*/*U*_eq_	Occ. (<1)
Ag1	0.313945 (8)	0.186742 (7)	0.040241 (15)	0.02197 (7)	
N1	0.25175 (10)	0.13210 (9)	−0.20778 (18)	0.0220 (4)	
N2	0.22372 (10)	0.08198 (8)	−0.07022 (18)	0.0219 (4)	
N3	0.39227 (10)	0.29823 (9)	0.12655 (19)	0.0236 (4)	
N4	0.37731 (10)	0.25291 (9)	0.27988 (18)	0.0227 (4)	
C1	0.25902 (11)	0.13002 (10)	−0.0883 (2)	0.0205 (4)	
C2	0.21334 (13)	0.08620 (10)	−0.2628 (2)	0.0250 (4)	
H2A	0.2020	0.0785	−0.3437	0.030*	
C3	0.19529 (12)	0.05430 (10)	−0.1766 (2)	0.0250 (4)	
H3A	0.1690	0.0205	−0.1866	0.030*	
C4	0.22223 (13)	0.05954 (11)	0.0474 (2)	0.0257 (4)	
H4A	0.1822	0.0348	0.0414	0.031*	
H4B	0.2180	0.0917	0.0994	0.031*	
C5	0.28652 (13)	0.02546 (10)	0.1002 (2)	0.0240 (4)	
C6	0.3103 (2)	−0.01707 (15)	0.0352 (3)	0.0424 (8)	
H6A	0.2872	−0.0240	−0.0429	0.051*	
C7	0.3684 (2)	−0.04945 (18)	0.0855 (3)	0.0580 (11)	
H7A	0.3843	−0.0777	0.0409	0.070*	
C8	0.40217 (19)	−0.04004 (17)	0.1999 (3)	0.0491 (9)	
H8A	0.4405	−0.0624	0.2337	0.059*	
C9	0.37995 (17)	0.00225 (15)	0.2653 (3)	0.0430 (7)	
H9A	0.4035	0.0088	0.3432	0.052*	
C10	0.32197 (15)	0.03570 (12)	0.2157 (2)	0.0310 (5)	
H10A	0.3073	0.0647	0.2602	0.037*	
C11	0.28146 (14)	0.17678 (12)	−0.2715 (2)	0.0288 (5)	
H11A	0.2954	0.2095	−0.2203	0.043*	
H11B	0.2476	0.1889	−0.3401	0.043*	
H11C	0.3208	0.1612	−0.2957	0.043*	
C12	0.36699 (11)	0.24814 (10)	0.1601 (2)	0.0210 (4)	
C13	0.41715 (12)	0.33379 (11)	0.2217 (2)	0.0272 (5)	
H13A	0.4363	0.3705	0.2196	0.033*	
C14	0.40833 (14)	0.30508 (12)	0.3196 (2)	0.0281 (5)	
H14A	0.4207	0.3179	0.3977	0.034*	
C15	0.36321 (12)	0.20739 (12)	0.3596 (2)	0.0267 (5)	
H15A	0.3339	0.1780	0.3138	0.032*	
H15B	0.3389	0.2240	0.4154	0.032*	
C16	0.42893 (13)	0.17983 (11)	0.4265 (2)	0.0242 (4)	
C17	0.45994 (14)	0.19764 (13)	0.5417 (2)	0.0303 (5)	
H17A	0.4396	0.2264	0.5786	0.036*	
C18	0.52170 (15)	0.17182 (16)	0.6012 (3)	0.0367 (6)	
H18A	0.5429	0.1843	0.6771	0.044*	
C19	0.55127 (14)	0.12872 (14)	0.5493 (3)	0.0362 (6)	
H19A	0.5918	0.1114	0.5906	0.043*	
C20	0.52082 (16)	0.11032 (13)	0.4341 (3)	0.0364 (6)	
H20A	0.5413	0.0812	0.3982	0.044*	
C21	0.45960 (14)	0.13608 (12)	0.3739 (2)	0.0304 (5)	
H21A	0.4390	0.1238	0.2976	0.036*	
C22	0.39464 (16)	0.31203 (12)	0.0039 (3)	0.0310 (5)	
H22A	0.3519	0.3009	−0.0483	0.046*	
H22B	0.4319	0.2912	−0.0171	0.046*	
H22C	0.4017	0.3530	−0.0032	0.046*	
K1	0.0000	0.08540 (3)	0.2500	0.02658 (15)	
P1	0.16091 (3)	0.15499 (3)	0.34572 (6)	0.02586 (13)	
F1	0.1121 (3)	0.1369 (2)	0.4315 (4)	0.0663 (14)	0.767 (17)
F2	0.2272 (4)	0.1299 (3)	0.4279 (6)	0.079 (2)	0.767 (17)
F3	0.1443 (3)	0.0957 (2)	0.2724 (5)	0.0475 (11)	0.767 (17)
F4	0.1743 (3)	0.2155 (3)	0.4111 (6)	0.074 (2)	0.767 (17)
F5	0.09354 (17)	0.18170 (17)	0.2567 (5)	0.0448 (9)	0.767 (17)
F6	0.20487 (19)	0.1757 (3)	0.2508 (3)	0.0307 (8)	0.767 (17)
F1A	0.1259 (10)	0.1383 (9)	0.4538 (18)	0.065 (4)*	0.233 (17)
F2A	0.2363 (8)	0.1276 (6)	0.4325 (14)	0.035 (3)*	0.233 (17)
F3A	0.1448 (7)	0.0905 (6)	0.2965 (13)	0.027 (2)*	0.233 (17)
F4A	0.1841 (8)	0.2166 (5)	0.4153 (12)	0.029 (3)*	0.233 (17)
F5A	0.0936 (7)	0.1771 (6)	0.2895 (16)	0.043 (3)*	0.233 (17)
F6A	0.2099 (8)	0.1626 (7)	0.2645 (16)	0.045 (4)*	0.233 (17)
P2	0.0000	0.0000	0.0000	0.02900 (19)	
F7	0.00552 (14)	0.06866 (9)	0.0087 (2)	0.0535 (5)	
F8	−0.05948 (12)	0.00366 (12)	−0.1174 (2)	0.0589 (6)	
F9	−0.05557 (11)	0.00116 (10)	0.0805 (2)	0.0524 (6)	

### Atomic displacement parameters (Å^2^)

**Table:**

	*U*^11^	*U*^22^	*U*^33^	*U*^12^	*U*^13^	*U*^23^
Ag1	0.02133 (11)	0.02546 (11)	0.01764 (11)	−0.00150 (5)	0.00131 (7)	−0.00177 (5)
N1	0.0217 (8)	0.0241 (9)	0.0194 (8)	0.0014 (7)	0.0034 (7)	0.0010 (7)
N2	0.0204 (8)	0.0223 (8)	0.0225 (9)	−0.0001 (7)	0.0039 (7)	−0.0005 (7)
N3	0.0211 (8)	0.0259 (9)	0.0222 (9)	0.0023 (7)	0.0014 (7)	0.0023 (7)
N4	0.0189 (8)	0.0283 (9)	0.0195 (9)	0.0010 (7)	0.0013 (7)	−0.0030 (7)
C1	0.0188 (9)	0.0221 (9)	0.0192 (9)	0.0011 (7)	0.0017 (7)	−0.0002 (7)
C2	0.0263 (10)	0.0251 (10)	0.0211 (10)	0.0029 (8)	0.0005 (8)	−0.0038 (8)
C3	0.0242 (10)	0.0233 (10)	0.0252 (11)	−0.0003 (8)	0.0009 (8)	−0.0034 (8)
C4	0.0250 (10)	0.0291 (11)	0.0250 (11)	0.0018 (8)	0.0100 (9)	0.0034 (9)
C5	0.0291 (11)	0.0210 (9)	0.0238 (10)	−0.0004 (8)	0.0100 (9)	0.0043 (8)
C6	0.067 (2)	0.0360 (15)	0.0256 (13)	0.0220 (14)	0.0136 (14)	0.0044 (10)
C7	0.086 (3)	0.053 (2)	0.0436 (18)	0.044 (2)	0.0335 (19)	0.0202 (16)
C8	0.0438 (16)	0.0560 (19)	0.0522 (19)	0.0212 (15)	0.0211 (15)	0.0304 (16)
C9	0.0385 (15)	0.0455 (16)	0.0392 (16)	−0.0030 (12)	−0.0034 (12)	0.0127 (13)
C10	0.0387 (13)	0.0263 (11)	0.0262 (12)	−0.0012 (10)	0.0034 (10)	0.0015 (9)
C11	0.0297 (12)	0.0323 (11)	0.0246 (11)	−0.0016 (9)	0.0066 (9)	0.0052 (9)
C12	0.0179 (9)	0.0263 (10)	0.0172 (9)	0.0019 (7)	0.0006 (7)	−0.0015 (7)
C13	0.0213 (10)	0.0249 (10)	0.0334 (12)	0.0011 (8)	0.0021 (9)	−0.0057 (9)
C14	0.0254 (11)	0.0308 (11)	0.0249 (11)	0.0011 (9)	−0.0009 (9)	−0.0081 (9)
C15	0.0203 (10)	0.0397 (13)	0.0199 (10)	−0.0002 (9)	0.0041 (8)	0.0011 (9)
C16	0.0216 (10)	0.0307 (11)	0.0197 (10)	−0.0015 (8)	0.0032 (8)	0.0039 (8)
C17	0.0277 (12)	0.0409 (13)	0.0209 (11)	0.0024 (10)	0.0029 (9)	0.0006 (10)
C18	0.0289 (12)	0.0556 (17)	0.0216 (12)	−0.0011 (12)	−0.0028 (10)	0.0072 (11)
C19	0.0235 (11)	0.0468 (15)	0.0384 (14)	0.0035 (10)	0.0071 (10)	0.0213 (12)
C20	0.0362 (14)	0.0366 (13)	0.0395 (15)	0.0091 (11)	0.0150 (12)	0.0105 (11)
C21	0.0306 (12)	0.0355 (13)	0.0256 (11)	−0.0010 (10)	0.0079 (9)	0.0004 (9)
C22	0.0321 (13)	0.0351 (13)	0.0261 (12)	0.0015 (9)	0.0073 (10)	0.0070 (9)
K1	0.0297 (3)	0.0282 (3)	0.0220 (3)	0.000	0.0061 (3)	0.000
P1	0.0312 (3)	0.0280 (3)	0.0191 (3)	−0.0048 (2)	0.0072 (2)	−0.0045 (2)
F1	0.100 (3)	0.076 (2)	0.0405 (19)	−0.052 (2)	0.053 (2)	−0.0271 (16)
F2	0.069 (3)	0.101 (4)	0.049 (2)	0.005 (2)	−0.023 (2)	0.026 (2)
F3	0.066 (2)	0.0377 (17)	0.047 (2)	−0.0194 (14)	0.0316 (19)	−0.0191 (17)
F4	0.079 (4)	0.066 (2)	0.092 (3)	−0.042 (2)	0.050 (3)	−0.057 (3)
F5	0.0300 (13)	0.065 (2)	0.0393 (19)	0.0101 (10)	0.0067 (13)	0.0182 (15)
F6	0.0294 (13)	0.0433 (19)	0.0212 (12)	−0.0078 (12)	0.0091 (9)	0.0043 (12)
P2	0.0251 (4)	0.0345 (5)	0.0291 (4)	−0.0088 (3)	0.0096 (3)	−0.0127 (3)
F7	0.0752 (15)	0.0358 (9)	0.0569 (13)	−0.0114 (9)	0.0306 (11)	−0.0140 (9)
F8	0.0468 (12)	0.0796 (16)	0.0428 (11)	0.0010 (11)	−0.0054 (9)	−0.0118 (11)
F9	0.0472 (11)	0.0645 (13)	0.0559 (12)	−0.0226 (10)	0.0333 (10)	−0.0300 (10)

### Geometric parameters (Å, °)

**Table:**

Ag1—C12	2.092 (2)	C17—H17A	0.9300
Ag1—C1	2.093 (2)	C18—C19	1.361 (5)
N1—C1	1.359 (3)	C18—H18A	0.9300
N1—C2	1.374 (3)	C19—C20	1.400 (5)
N1—C11	1.467 (3)	C19—H19A	0.9300
N2—C1	1.353 (3)	C20—C21	1.393 (4)
N2—C3	1.389 (3)	C20—H20A	0.9300
N2—C4	1.463 (3)	C21—H21A	0.9300
N3—C12	1.351 (3)	C22—H22A	0.9600
N3—C13	1.372 (3)	C22—H22B	0.9600
N3—C22	1.467 (3)	C22—H22C	0.9600
N4—C12	1.360 (3)	K1—F5A^i^	2.786 (14)
N4—C14	1.382 (3)	K1—F5A	2.786 (14)
N4—C15	1.466 (3)	K1—F9	2.803 (2)
C2—C3	1.352 (4)	K1—F9^i^	2.803 (2)
C2—H2A	0.9300	K1—F3A^i^	2.813 (13)
C3—H3A	0.9300	K1—F3A	2.813 (13)
C4—C5	1.509 (3)	K1—F3	2.836 (5)
C4—H4A	0.9700	K1—F3^i^	2.836 (5)
C4—H4B	0.9700	K1—F7^i^	2.848 (2)
C5—C6	1.383 (4)	K1—F7	2.848 (2)
C5—C10	1.384 (4)	K1—F5	2.887 (4)
C6—C7	1.390 (5)	K1—F5^i^	2.887 (4)
C6—H6A	0.9300	P1—F5A	1.444 (14)
C7—C8	1.359 (6)	P1—F6A	1.512 (16)
C7—H7A	0.9300	P1—F2	1.554 (5)
C8—C9	1.368 (6)	P1—F4	1.580 (5)
C8—H8A	0.9300	P1—F1	1.596 (3)
C9—C10	1.399 (4)	P1—F3A	1.597 (14)
C9—H9A	0.9300	P1—F3	1.604 (4)
C10—H10A	0.9300	P1—F1A	1.612 (17)
C11—H11A	0.9600	P1—F5	1.617 (4)
C11—H11B	0.9600	P1—F6	1.625 (3)
C11—H11C	0.9600	P1—F4A	1.646 (14)
C13—C14	1.358 (4)	P1—F2A	1.725 (16)
C13—H13A	0.9300	P2—F7	1.588 (2)
C14—H14A	0.9300	P2—F7^ii^	1.588 (2)
C15—C16	1.502 (3)	P2—F8	1.590 (2)
C15—H15A	0.9700	P2—F8^ii^	1.590 (2)
C15—H15B	0.9700	P2—F9	1.6010 (18)
C16—C21	1.389 (4)	P2—F9^ii^	1.6010 (18)
C16—C17	1.399 (4)	P2—K1^ii^	3.5004 (5)
C17—C18	1.399 (4)	F8—K1^ii^	2.966 (3)
			
C12—Ag1—C1	175.99 (9)	F5A^i^—K1—F7	96.6 (4)
C1—N1—C2	111.4 (2)	F5A—K1—F7	95.2 (4)
C1—N1—C11	125.1 (2)	F9—K1—F7	46.86 (6)
C2—N1—C11	123.6 (2)	F9^i^—K1—F7	119.74 (7)
C1—N2—C3	111.1 (2)	F3A^i^—K1—F7	94.8 (3)
C1—N2—C4	123.6 (2)	F3A—K1—F7	85.8 (3)
C3—N2—C4	124.9 (2)	F3—K1—F7	80.62 (12)
C12—N3—C13	111.7 (2)	F3^i^—K1—F7	100.69 (13)
C12—N3—C22	124.0 (2)	F7^i^—K1—F7	164.42 (9)
C13—N3—C22	124.3 (2)	F5A^i^—K1—F5	79.9 (2)
C12—N4—C14	111.2 (2)	F5A—K1—F5	7.7 (3)
C12—N4—C15	125.3 (2)	F9—K1—F5	134.50 (12)
C14—N4—C15	123.2 (2)	F9^i^—K1—F5	111.32 (10)
N2—C1—N1	104.30 (19)	F3A^i^—K1—F5	126.9 (3)
N2—C1—Ag1	127.39 (17)	F3A—K1—F5	48.3 (3)
N1—C1—Ag1	128.29 (17)	F3—K1—F5	45.51 (12)
C3—C2—N1	106.8 (2)	F3^i^—K1—F5	124.94 (13)
C3—C2—H2A	126.6	F7^i^—K1—F5	104.25 (12)
N1—C2—H2A	126.6	F7—K1—F5	87.83 (12)
C2—C3—N2	106.4 (2)	F5A^i^—K1—F5^i^	7.7 (3)
C2—C3—H3A	126.8	F5A—K1—F5^i^	79.9 (2)
N2—C3—H3A	126.8	F9—K1—F5^i^	111.32 (10)
N2—C4—C5	111.51 (19)	F9^i^—K1—F5^i^	134.51 (12)
N2—C4—H4A	109.3	F3A^i^—K1—F5^i^	48.3 (3)
C5—C4—H4A	109.3	F3A—K1—F5^i^	126.9 (3)
N2—C4—H4B	109.3	F3—K1—F5^i^	124.94 (13)
C5—C4—H4B	109.3	F3^i^—K1—F5^i^	45.51 (12)
H4A—C4—H4B	108.0	F7^i^—K1—F5^i^	87.83 (12)
C6—C5—C10	119.0 (3)	F7—K1—F5^i^	104.25 (12)
C6—C5—C4	120.8 (2)	F5—K1—F5^i^	79.54 (13)
C10—C5—C4	120.2 (2)	F5A—P1—F6A	110.1 (11)
C5—C6—C7	120.5 (3)	F5A—P1—F2	168.8 (7)
C5—C6—H6A	119.8	F6A—P1—F2	81.1 (8)
C7—C6—H6A	119.8	F5A—P1—F4	86.8 (6)
C8—C7—C6	120.3 (3)	F6A—P1—F4	97.7 (7)
C8—C7—H7A	119.9	F2—P1—F4	90.4 (4)
C6—C7—H7A	119.9	F5A—P1—F1	74.8 (7)
C7—C8—C9	120.2 (3)	F6A—P1—F1	171.4 (7)
C7—C8—H8A	119.9	F2—P1—F1	94.3 (4)
C9—C8—H8A	119.9	F4—P1—F1	89.6 (3)
C8—C9—C10	120.4 (3)	F5A—P1—F3A	94.3 (7)
C8—C9—H9A	119.8	F6A—P1—F3A	89.5 (8)
C10—C9—H9A	119.8	F2—P1—F3A	87.0 (6)
C5—C10—C9	119.7 (3)	F4—P1—F3A	171.9 (6)
C5—C10—H10A	120.1	F1—P1—F3A	83.0 (6)
C9—C10—H10A	120.1	F5A—P1—F3	90.2 (6)
N1—C11—H11A	109.5	F6A—P1—F3	81.6 (7)
N1—C11—H11B	109.5	F2—P1—F3	93.0 (4)
H11A—C11—H11B	109.5	F4—P1—F3	176.4 (4)
N1—C11—H11C	109.5	F1—P1—F3	91.4 (2)
H11A—C11—H11C	109.5	F5A—P1—F1A	85.1 (8)
H11B—C11—H11C	109.5	F6A—P1—F1A	164.8 (11)
N3—C12—N4	104.1 (2)	F2—P1—F1A	83.9 (8)
N3—C12—Ag1	123.54 (16)	F4—P1—F1A	83.9 (8)
N4—C12—Ag1	131.89 (17)	F3A—P1—F1A	88.1 (8)
C14—C13—N3	106.7 (2)	F3—P1—F1A	97.7 (8)
C14—C13—H13A	126.6	F6A—P1—F5	96.8 (8)
N3—C13—H13A	126.6	F2—P1—F5	177.9 (3)
C13—C14—N4	106.2 (2)	F4—P1—F5	89.7 (3)
C13—C14—H14A	126.9	F1—P1—F5	87.8 (3)
N4—C14—H14A	126.9	F3A—P1—F5	93.2 (5)
N4—C15—C16	110.98 (19)	F3—P1—F5	86.9 (3)
N4—C15—H15A	109.4	F1A—P1—F5	98.3 (7)
C16—C15—H15A	109.4	F5A—P1—F6	100.3 (8)
N4—C15—H15B	109.4	F2—P1—F6	90.6 (4)
C16—C15—H15B	109.4	F4—P1—F6	90.4 (3)
H15A—C15—H15B	108.0	F1—P1—F6	175.1 (4)
C21—C16—C17	119.2 (2)	F3A—P1—F6	97.3 (5)
C21—C16—C15	120.0 (2)	F3—P1—F6	88.3 (2)
C17—C16—C15	120.8 (2)	F1A—P1—F6	172.0 (8)
C18—C17—C16	119.6 (3)	F5—P1—F6	87.3 (3)
C18—C17—H17A	120.2	F5A—P1—F4A	92.6 (7)
C16—C17—H17A	120.2	F6A—P1—F4A	93.2 (8)
C19—C18—C17	120.9 (3)	F2—P1—F4A	85.2 (5)
C19—C18—H18A	119.6	F1—P1—F4A	93.6 (6)
C17—C18—H18A	119.6	F3A—P1—F4A	171.2 (7)
C18—C19—C20	120.3 (3)	F3—P1—F4A	174.7 (6)
C18—C19—H19A	119.9	F1A—P1—F4A	87.1 (9)
C20—C19—H19A	119.9	F5—P1—F4A	94.8 (4)
C21—C20—C19	119.2 (3)	F6—P1—F4A	86.8 (6)
C21—C20—H20A	120.4	F5A—P1—F2A	171.1 (9)
C19—C20—H20A	120.4	F6A—P1—F2A	78.8 (8)
C16—C21—C20	120.8 (3)	F4—P1—F2A	90.8 (5)
C16—C21—H21A	119.6	F1—P1—F2A	96.6 (6)
C20—C21—H21A	119.6	F3A—P1—F2A	86.9 (6)
N3—C22—H22A	109.5	F3—P1—F2A	92.5 (5)
N3—C22—H22B	109.5	F1A—P1—F2A	86.2 (8)
H22A—C22—H22B	109.5	F5—P1—F2A	175.6 (5)
N3—C22—H22C	109.5	F6—P1—F2A	88.3 (5)
H22A—C22—H22C	109.5	F4A—P1—F2A	85.4 (6)
H22B—C22—H22C	109.5	P1—F1—K1	97.5 (2)
F5A^i^—K1—F5A	81.3 (6)	P1—F3—K1	101.7 (2)
F5A^i^—K1—F9	105.5 (3)	P1—F5—K1	99.30 (19)
F5A—K1—F9	141.6 (4)	P1—F1A—K1	85.7 (9)
F5A^i^—K1—F9^i^	141.6 (4)	P1—F3A—K1	102.8 (6)
F5A—K1—F9^i^	105.5 (3)	P1—F5A—K1	108.9 (7)
F9—K1—F9^i^	92.34 (12)	F7—P2—F7^ii^	180.0
F5A^i^—K1—F3A^i^	47.0 (4)	F7—P2—F8	91.64 (14)
F5A—K1—F3A^i^	128.2 (4)	F7^ii^—P2—F8	88.36 (14)
F9—K1—F3A^i^	70.8 (3)	F7—P2—F8^ii^	88.36 (14)
F9^i^—K1—F3A^i^	112.8 (3)	F7^ii^—P2—F8^ii^	91.64 (14)
F5A^i^—K1—F3A	128.2 (4)	F8—P2—F8^ii^	180.0
F5A—K1—F3A	47.0 (4)	F7—P2—F9	89.64 (12)
F9—K1—F3A	112.8 (3)	F7^ii^—P2—F9	90.36 (12)
F9^i^—K1—F3A	70.8 (3)	F8—P2—F9	91.02 (13)
F3A^i^—K1—F3A	175.2 (5)	F8^ii^—P2—F9	88.99 (13)
F5A^i^—K1—F3	125.3 (3)	F7—P2—F9^ii^	90.36 (12)
F5A—K1—F3	45.2 (3)	F7^ii^—P2—F9^ii^	89.64 (12)
F9—K1—F3	110.65 (9)	F8—P2—F9^ii^	88.98 (13)
F9^i^—K1—F3	76.28 (14)	F8^ii^—P2—F9^ii^	91.02 (13)
F3A^i^—K1—F3	170.9 (3)	F9—P2—F9^ii^	179.999 (1)
F3A—K1—F3	6.1 (3)	F7—P2—K1^ii^	126.84 (8)
F5A^i^—K1—F3^i^	45.2 (3)	F7^ii^—P2—K1^ii^	53.16 (8)
F5A—K1—F3^i^	125.3 (3)	F8—P2—K1^ii^	57.49 (9)
F9—K1—F3^i^	76.28 (14)	F8^ii^—P2—K1^ii^	122.51 (9)
F9^i^—K1—F3^i^	110.65 (9)	F9—P2—K1^ii^	128.37 (7)
F3A^i^—K1—F3^i^	6.1 (3)	F9^ii^—P2—K1^ii^	51.63 (7)
F3A—K1—F3^i^	170.9 (3)	F7—P2—K1	53.16 (8)
F3—K1—F3^i^	170.4 (2)	F7^ii^—P2—K1	126.84 (8)
F5A^i^—K1—F7^i^	95.2 (4)	F8—P2—K1	122.51 (9)
F5A—K1—F7^i^	96.6 (4)	F8^ii^—P2—K1	57.49 (9)
F9—K1—F7^i^	119.74 (7)	F9—P2—K1	51.63 (7)
F9^i^—K1—F7^i^	46.87 (6)	F9^ii^—P2—K1	128.37 (7)
F3A^i^—K1—F7^i^	85.8 (3)	K1^ii^—P2—K1	180.0
F3A—K1—F7^i^	94.8 (3)	P2—F7—K1	100.34 (11)
F3—K1—F7^i^	100.69 (13)	P2—F8—K1^ii^	95.64 (11)
F3^i^—K1—F7^i^	80.62 (12)	P2—F9—K1	101.77 (9)
			
C3—N2—C1—N1	0.4 (2)	F5—P1—F3A—K1	−36.3 (6)
C4—N2—C1—N1	174.6 (2)	F6—P1—F3A—K1	−124.0 (4)
C3—N2—C1—Ag1	−178.37 (16)	F2A—P1—F3A—K1	148.1 (7)
C4—N2—C1—Ag1	−4.2 (3)	F5A^i^—K1—F3A—P1	22.0 (8)
C2—N1—C1—N2	−0.5 (2)	F5A—K1—F3A—P1	16.0 (7)
C11—N1—C1—N2	−179.7 (2)	F9—K1—F3A—P1	156.4 (5)
C2—N1—C1—Ag1	178.26 (16)	F9^i^—K1—F3A—P1	−119.4 (7)
C11—N1—C1—Ag1	−1.0 (3)	F3—K1—F3A—P1	86 (3)
C1—N1—C2—C3	0.4 (3)	F7^i^—K1—F3A—P1	−78.5 (6)
C11—N1—C2—C3	179.6 (2)	F7—K1—F3A—P1	117.1 (6)
N1—C2—C3—N2	−0.1 (3)	F5—K1—F3A—P1	26.3 (5)
C1—N2—C3—C2	−0.2 (3)	F5^i^—K1—F3A—P1	12.4 (8)
C4—N2—C3—C2	−174.3 (2)	F6A—P1—F5A—K1	115.1 (10)
C1—N2—C4—C5	−79.8 (3)	F2—P1—F5A—K1	−72 (3)
C3—N2—C4—C5	93.6 (3)	F4—P1—F5A—K1	−147.9 (7)
N2—C4—C5—C6	−48.9 (3)	F1—P1—F5A—K1	−57.5 (6)
N2—C4—C5—C10	132.3 (2)	F3A—P1—F5A—K1	24.0 (10)
C10—C5—C6—C7	0.8 (5)	F3—P1—F5A—K1	34.0 (7)
C4—C5—C6—C7	−178.1 (3)	F1A—P1—F5A—K1	−63.7 (11)
C5—C6—C7—C8	0.6 (6)	F5—P1—F5A—K1	109 (3)
C6—C7—C8—C9	−1.4 (6)	F6—P1—F5A—K1	122.2 (7)
C7—C8—C9—C10	0.7 (6)	F4A—P1—F5A—K1	−150.6 (8)
C6—C5—C10—C9	−1.4 (4)	F5A^i^—K1—F5A—P1	166.3 (13)
C4—C5—C10—C9	177.5 (3)	F9—K1—F5A—P1	−89.6 (10)
C8—C9—C10—C5	0.7 (5)	F9^i^—K1—F5A—P1	25.1 (9)
C13—N3—C12—N4	−0.6 (3)	F3A^i^—K1—F5A—P1	162.0 (8)
C22—N3—C12—N4	177.7 (2)	F3—K1—F5A—P1	−26.5 (6)
C13—N3—C12—Ag1	172.52 (16)	F3^i^—K1—F5A—P1	155.2 (7)
C22—N3—C12—Ag1	−9.2 (3)	F7^i^—K1—F5A—P1	72.1 (9)
C14—N4—C12—N3	0.1 (3)	F7—K1—F5A—P1	−97.8 (9)
C15—N4—C12—N3	−175.1 (2)	F5—K1—F5A—P1	−115 (3)
C14—N4—C12—Ag1	−172.17 (18)	F5^i^—K1—F5A—P1	158.7 (10)
C15—N4—C12—Ag1	12.6 (3)	F5A^i^—K1—P2—F7	50.6 (3)
C12—N3—C13—C14	0.8 (3)	F5A—K1—P2—F7	−41.6 (4)
C22—N3—C13—C14	−177.4 (2)	F9—K1—P2—F7	125.66 (18)
N3—C13—C14—N4	−0.7 (3)	F9^i^—K1—P2—F7	−152.39 (13)
C12—N4—C14—C13	0.4 (3)	F3A^i^—K1—P2—F7	94.2 (3)
C15—N4—C14—C13	175.7 (2)	F3A—K1—P2—F7	−81.8 (3)
C12—N4—C15—C16	105.0 (3)	F3—K1—P2—F7	−76.70 (18)
C14—N4—C15—C16	−69.7 (3)	F3^i^—K1—P2—F7	95.36 (15)
N4—C15—C16—C21	−82.8 (3)	F7^i^—K1—P2—F7	−178.38 (4)
N4—C15—C16—C17	97.3 (3)	F5—K1—P2—F7	−36.96 (14)
C21—C16—C17—C18	1.0 (4)	F5^i^—K1—P2—F7	52.71 (15)
C15—C16—C17—C18	−179.0 (3)	F5A^i^—K1—P2—F7^ii^	−129.4 (3)
C16—C17—C18—C19	−1.4 (4)	F5A—K1—P2—F7^ii^	138.4 (4)
C17—C18—C19—C20	1.3 (5)	F9—K1—P2—F7^ii^	−54.34 (18)
C18—C19—C20—C21	−0.8 (4)	F9^i^—K1—P2—F7^ii^	27.61 (13)
C17—C16—C21—C20	−0.6 (4)	F3A^i^—K1—P2—F7^ii^	−85.8 (3)
C15—C16—C21—C20	179.5 (2)	F3A—K1—P2—F7^ii^	98.2 (3)
C19—C20—C21—C16	0.4 (4)	F3—K1—P2—F7^ii^	103.30 (18)
F5A—P1—F1—K1	49.5 (6)	F3^i^—K1—P2—F7^ii^	−84.64 (15)
F2—P1—F1—K1	−133.3 (3)	F7^i^—K1—P2—F7^ii^	1.62 (4)
F4—P1—F1—K1	136.3 (3)	F7—K1—P2—F7^ii^	180.0
F3A—P1—F1—K1	−46.9 (5)	F5—K1—P2—F7^ii^	143.03 (14)
F3—P1—F1—K1	−40.2 (3)	F5^i^—K1—P2—F7^ii^	−127.29 (15)
F1A—P1—F1—K1	−162 (4)	F5A^i^—K1—P2—F8	−13.6 (3)
F5—P1—F1—K1	46.6 (2)	F5A—K1—P2—F8	−105.8 (4)
F4A—P1—F1—K1	141.2 (4)	F9—K1—P2—F8	61.46 (18)
F2A—P1—F1—K1	−133.0 (5)	F3A^i^—K1—P2—F8	30.0 (3)
F5A^i^—K1—F1—P1	−99.2 (5)	F3A—K1—P2—F8	−146.0 (3)
F5A—K1—F1—P1	−38.6 (5)	F3—K1—P2—F8	−140.91 (18)
F9—K1—F1—P1	67.2 (4)	F3^i^—K1—P2—F8	31.15 (15)
F9^i^—K1—F1—P1	119.6 (3)	F7^i^—K1—P2—F8	117.41 (15)
F3A^i^—K1—F1—P1	−137.2 (5)	F7—K1—P2—F8	−64.20 (17)
F3A—K1—F1—P1	37.1 (5)	F5—K1—P2—F8	−101.17 (15)
F3—K1—F1—P1	30.2 (3)	F5^i^—K1—P2—F8	−11.50 (15)
F3^i^—K1—F1—P1	−139.1 (2)	F5A^i^—K1—P2—F8^ii^	166.4 (3)
F7^i^—K1—F1—P1	170.3 (3)	F5A—K1—P2—F8^ii^	74.2 (4)
F7—K1—F1—P1	6.3 (4)	F9—K1—P2—F8^ii^	−118.54 (18)
F5—K1—F1—P1	−35.15 (18)	F9^i^—K1—P2—F8^ii^	−36.60 (13)
F5^i^—K1—F1—P1	−104.8 (3)	F3A^i^—K1—P2—F8^ii^	−150.0 (3)
F5A—P1—F3—K1	−32.0 (8)	F3A—K1—P2—F8^ii^	34.0 (3)
F6A—P1—F3—K1	−142.3 (6)	F3—K1—P2—F8^ii^	39.09 (18)
F2—P1—F3—K1	137.2 (4)	F3^i^—K1—P2—F8^ii^	−148.85 (15)
F1—P1—F3—K1	42.8 (4)	F7^i^—K1—P2—F8^ii^	−62.59 (15)
F3A—P1—F3—K1	81 (3)	F7—K1—P2—F8^ii^	115.79 (17)
F1A—P1—F3—K1	53.0 (8)	F5—K1—P2—F8^ii^	78.83 (15)
F5—P1—F3—K1	−44.9 (3)	F5^i^—K1—P2—F8^ii^	168.50 (15)
F6—P1—F3—K1	−132.3 (2)	F5A—K1—P2—F9	−167.2 (4)
F2A—P1—F3—K1	139.5 (5)	F9^i^—K1—P2—F9	81.94 (16)
F5A^i^—K1—F3—P1	38.3 (4)	F3A^i^—K1—P2—F9	−31.5 (3)
F5A—K1—F3—P1	22.8 (6)	F3A—K1—P2—F9	152.5 (3)
F9—K1—F3—P1	166.4 (2)	F3—K1—P2—F9	157.63 (19)
F9^i^—K1—F3—P1	−106.3 (3)	F3^i^—K1—P2—F9	−30.31 (15)
F3A—K1—F3—P1	−82 (3)	F7^i^—K1—P2—F9	55.95 (15)
F7^i^—K1—F3—P1	−65.9 (3)	F7—K1—P2—F9	−125.66 (18)
F7—K1—F3—P1	129.8 (3)	F5—K1—P2—F9	−162.63 (15)
F5—K1—F3—P1	33.6 (3)	F5^i^—K1—P2—F9	−72.96 (16)
F5^i^—K1—F3—P1	28.9 (3)	F5A^i^—K1—P2—F9^ii^	105.0 (3)
F5A—P1—F5—K1	−61 (3)	F5A—K1—P2—F9^ii^	12.8 (4)
F6A—P1—F5—K1	124.6 (7)	F9—K1—P2—F9^ii^	180.0
F4—P1—F5—K1	−137.7 (3)	F9^i^—K1—P2—F9^ii^	−98.06 (16)
F1—P1—F5—K1	−48.1 (2)	F3A^i^—K1—P2—F9^ii^	148.5 (3)
F3A—P1—F5—K1	34.7 (6)	F3A—K1—P2—F9^ii^	−27.5 (3)
F3—P1—F5—K1	43.5 (3)	F3—K1—P2—F9^ii^	−22.37 (19)
F1A—P1—F5—K1	−53.8 (8)	F3^i^—K1—P2—F9^ii^	149.69 (15)
F6—P1—F5—K1	131.9 (3)	F7^i^—K1—P2—F9^ii^	−124.05 (15)
F4A—P1—F5—K1	−141.6 (6)	F7—K1—P2—F9^ii^	54.33 (18)
F5A^i^—K1—F5—P1	150.9 (5)	F5—K1—P2—F9^ii^	17.37 (15)
F5A—K1—F5—P1	51 (2)	F5^i^—K1—P2—F9^ii^	107.04 (16)
F9—K1—F5—P1	−107.2 (2)	F8—P2—F7—K1	130.58 (12)
F9^i^—K1—F5—P1	9.2 (2)	F8^ii^—P2—F7—K1	−49.43 (12)
F3A^i^—K1—F5—P1	153.6 (4)	F9—P2—F7—K1	39.57 (12)
F3A—K1—F5—P1	−25.6 (5)	F9^ii^—P2—F7—K1	−140.43 (12)
F3—K1—F5—P1	−33.0 (2)	K1^ii^—P2—F7—K1	180.0
F3^i^—K1—F5—P1	146.5 (2)	F5A^i^—K1—F7—P2	−135.0 (3)
F7^i^—K1—F5—P1	58.1 (2)	F5A—K1—F7—P2	143.2 (3)
F7—K1—F5—P1	−112.0 (2)	F9—K1—F7—P2	−29.90 (10)
F5^i^—K1—F5—P1	143.1 (3)	F9^i^—K1—F7—P2	32.02 (15)
F5A—P1—F1A—K1	46.5 (6)	F3A^i^—K1—F7—P2	−87.7 (3)
F6A—P1—F1A—K1	−129 (4)	F3A—K1—F7—P2	97.1 (3)
F2—P1—F1A—K1	−135.1 (5)	F3—K1—F7—P2	100.23 (16)
F4—P1—F1A—K1	133.8 (4)	F3^i^—K1—F7—P2	−89.39 (15)
F1—P1—F1A—K1	16 (4)	F7^i^—K1—F7—P2	4.04 (10)
F3A—P1—F1A—K1	−48.0 (5)	F5—K1—F7—P2	145.43 (13)
F3—P1—F1A—K1	−43.0 (4)	F5^i^—K1—F7—P2	−135.93 (12)
F5—P1—F1A—K1	45.0 (4)	F7—P2—F8—K1^ii^	133.87 (11)
F4A—P1—F1A—K1	139.4 (5)	F7^ii^—P2—F8—K1^ii^	−46.13 (11)
F2A—P1—F1A—K1	−135.0 (6)	F9—P2—F8—K1^ii^	−136.46 (11)
F5A^i^—K1—F1A—P1	−95.8 (7)	F9^ii^—P2—F8—K1^ii^	43.54 (11)
F5A—K1—F1A—P1	−36.6 (6)	K1—P2—F8—K1^ii^	180.0
F9—K1—F1A—P1	72.8 (10)	F7—P2—F9—K1	−40.56 (13)
F9^i^—K1—F1A—P1	123.3 (7)	F7^ii^—P2—F9—K1	139.44 (13)
F3A^i^—K1—F1A—P1	−135.3 (6)	F8—P2—F9—K1	−132.19 (13)
F3A—K1—F1A—P1	38.8 (6)	F8^ii^—P2—F9—K1	47.81 (13)
F3—K1—F1A—P1	32.0 (4)	K1^ii^—P2—F9—K1	180.0
F3^i^—K1—F1A—P1	−136.9 (5)	F5A^i^—K1—F9—P2	114.3 (4)
F7^i^—K1—F1A—P1	174.7 (8)	F5A—K1—F9—P2	18.6 (5)
F7—K1—F1A—P1	11.1 (9)	F9^i^—K1—F9—P2	−100.15 (13)
F5—K1—F1A—P1	−32.8 (4)	F3A^i^—K1—F9—P2	146.5 (3)
F5^i^—K1—F1A—P1	−101.3 (7)	F3A—K1—F9—P2	−30.1 (3)
F5A—P1—F3A—K1	−23.0 (10)	F3—K1—F9—P2	−23.9 (2)
F6A—P1—F3A—K1	−133.1 (7)	F3^i^—K1—F9—P2	149.16 (15)
F2—P1—F3A—K1	145.8 (6)	F7^i^—K1—F9—P2	−140.27 (11)
F1—P1—F3A—K1	51.1 (6)	F7—K1—F9—P2	29.79 (10)
F3—P1—F3A—K1	−90 (3)	F5—K1—F9—P2	23.24 (19)
F1A—P1—F3A—K1	61.9 (9)		

Symmetry codes: (i) −*x*, *y*, −*z*+1/2; (ii) −*x*, −*y*, −*z*.

### Hydrogen-bond geometry (Å, °)

**Table:**

*D*—H···*A*	*D*—H	H···*A*	*D*···*A*	*D*—H···*A*
C14—H14A···F1^iii^	0.93	2.45	3.285 (5)	149
C15—H15A···F6	0.97	2.51	3.204 (5)	129
C15—H15B···F4^iii^	0.97	2.51	3.415 (7)	156
C22—H22A···F6^iv^	0.96	2.42	3.171 (5)	135

Symmetry codes: (iii) −*x*+1/2, −*y*+1/2, −*z*+1; (iv) −*x*+1/2, −*y*+1/2, −*z*.
